# Myocardial steatosis, impaired energetics and reduced circumferential strain are early manifestations of diabetic cardiomyopathy and precede structural changes

**DOI:** 10.1186/1532-429X-16-S1-O114

**Published:** 2014-01-16

**Authors:** Eylem Levelt, Masliza Mahmod, Camilla Wainwright, Stefan K Piechnik, Jane M Francis, Jurgen E Schneider, Paul Leeson, Theodoros D Karamitsos, Cameron Holloway, Kieran Clarke, Stefan Neubauer

**Affiliations:** 1 University of Oxford Centre for Clinical Magnetic Resonance Research, University of Oxford, Oxford, UK; 2 Department of Physiology, Anatomy and Genetics, University of Oxford, Oxford, UK; 3 Oxford Cardiovascular Clinical Research Facility, University of Oxford, Oxford, UK

## Background

Patients with diabetes mellitus (DM) of longer duration have an increased incidence of heart failure, independent of hypertension and coronary disease, as shown in large population based studies. Mechanisms of diabetic cardiomyopathy are multifactorial and not fully elucidated. Cardiovascular magnetic resonance (CMR) and magnetic resonance spectrocopy (MRS) provide a non-invasive assessment of the functional, structural and metabolic status of the heart. Here we assessed whether subclinical functional, structural and metabolic alterations can be detected using MRI in a patient cohort of early-onset, stable and uncomplicated type 2 DM.

## Objective

The aim of this study was to assess the earliest manifestations of diabetic cardiomyopathy using multiparametric CMR and MRS in a cohort of uncomplicated type 2 DM patients with a short duration of disease.

## Methods

13 patients (6 female, mean age 55 ± 9 years) with early-onset (median 3 [IQR: 1-4] years) type 2 DM and 10 healthy volunteers (5 female, mean age 57 ± 9 years) were enrolled. Patients were either drug naive for diabetic therapy or on treatment with metformin monotherapy, HBA1c ≥ 6.7 and ≤ 8%, with no history of coronary artery disease or uncontrolled hypertension. Myocardial lipid content and PCr/ATP ratios were quantified using 1H- and 31P MRS, respectively. CMR included cine, tagging and native T1 mapping was performed at 3.0 T. LV diastology was characterised using echocardiography.

## Results

**Figure 1:**
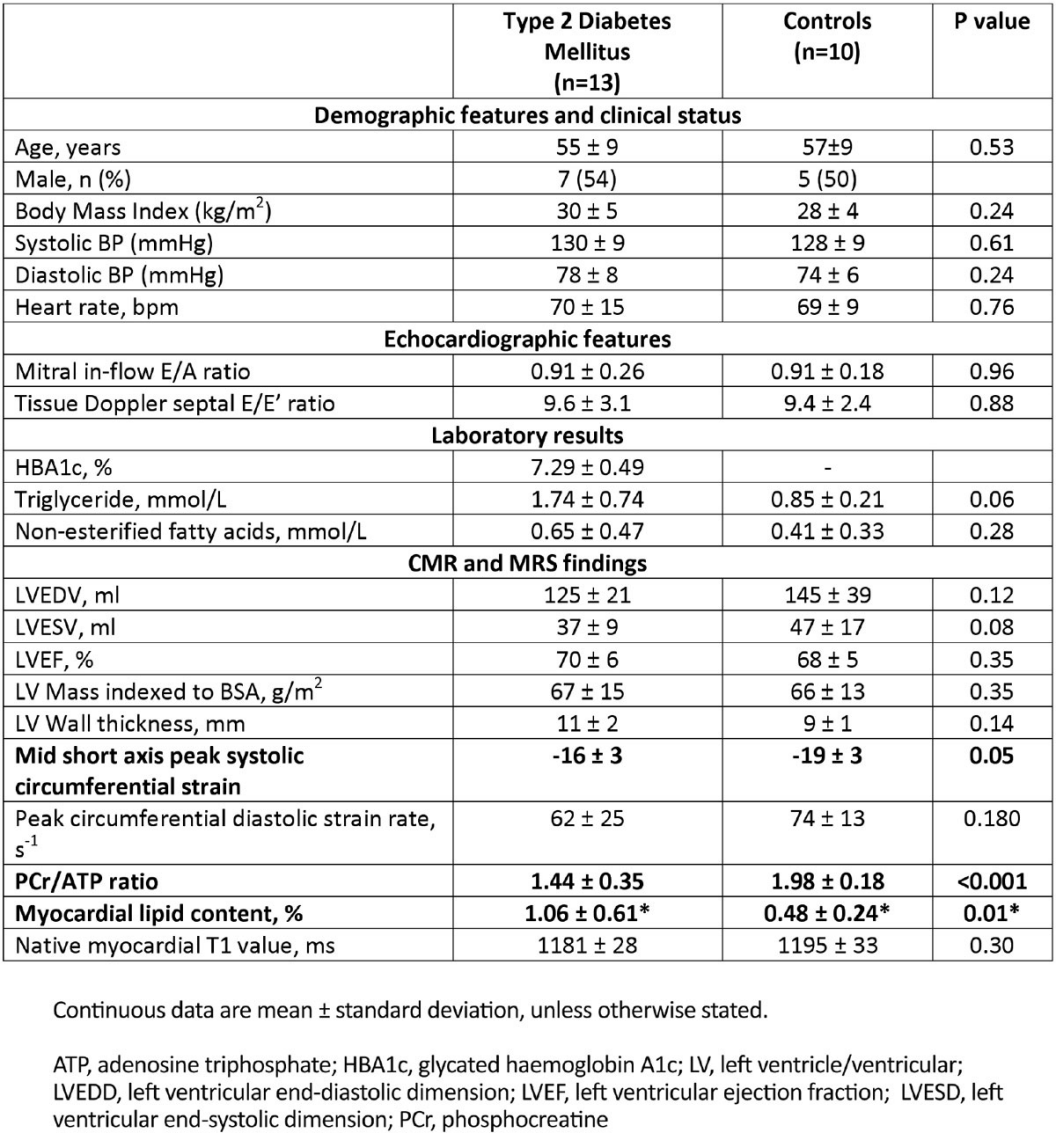


Diabetic patients were well-matched with controls (Figure 1). Myocardial energetics were disturbed (PCr/ATP ratio: 1.44 ± 0.35 vs. 1.98 ± 0.18, p < 0.001) and myocardial triglyceride content increased (1.06 ± 0.61 vs. 0.48 ± 0.24%, p = 0.01), despite the relatively short disease duration. Peak systolic circumferential strain was reduced, indicating subtle regional LV dysfunction. However, left ventricular volumes, mass and ejection fraction, as well as echocardiographic indices of diastolic function were similar in both groups. Furthermore, despite the metabolic abnormalities observed in diabetics, there was no difference in native T1 values (as a measure of myocardial fibrosis) between diabetic patients and controls (1181 ± 28 ms vs. 1195 ± 33 ms, p = 0.30).

## Conclusions

Abnormal myocardial energy metabolism, cardiac steatosis and reduced LV strain are present in uncomplicated type 2 DM patients with a short duration of disease and precede the development of structural or other functional changes. CMR is a sensitive, non-invasive tool for assessment of myocardial pathophysiology, and may be helpful in the comprehensive phenotyping and staging of myocardial involvement in DM.

## Funding

'The National Institute for Health Research Oxford Biomedical Research Council and Hoffmann-La Roche supported this work.

